# DL-3-n-Butylphthalide Attenuates Myocardial Hypertrophy by Targeting Gasdermin D and Inhibiting Gasdermin D Mediated Inflammation

**DOI:** 10.3389/fphar.2021.688140

**Published:** 2021-06-08

**Authors:** Bingjiang Han, Jiajun Xu, Xiaowen Shi, Zhanxiong Zheng, Fengjie Shi, Fenfen Jiang, Jibo Han

**Affiliations:** Department of Cardiology, The Second Affiliated Hospital of Jiaxing University, Jiaxing, China

**Keywords:** dl-3-n-butylphthalide, myocardial hypertrophy, GSDMD, inflammation, target

## Abstract

Pressure overload leads to a hypertrophic milieu that produces deleterious cardiac dysfunction. Inflammation is a key pathophysiological mechanism underpinning myocardial hypertrophy. DL-3-n-butylphthalide (NBP), a neuroprotective agent, also has potent cardioprotective effects. In this study, the potential of NBP to antagonize myocardial hypertrophy was evaluated in C57BL/6 mice *in vivo* and in rat primary cardiomyocytes *in vitro*. In mice, NBP treatment reduced cardiac hypertrophy and dysfunction in a transverse aortic constriction (TAC)-induced pressure overload model. In angiotensin (Ang) II-challenged cardiomyocytes, NBP prevents cell size increases and inhibits gasdermin D (GSDMD)-mediated inflammation. Furthermore, overexpression of GSDMD-N reduced the protective effects of NBP against Ang II-induced changes. Using molecular docking and MD simulation, we found that the GSDMD-N protein may be a target of NBP. Our study shows that NBP attenuates myocardial hypertrophy by targeting GSDMD and inhibiting GSDMD-mediated inflammation.

## Introduction

Hypertensive heart disease is characterized by adverse ventricular hypertrophy and cardiac dysfunction, ultimately leading to heart failure (Weber and Brilla, 1991). Experimental and clinical studies have reported that the inflammatory response plays an important role in myocardial hypertrophy (Freund et al., 2005). Pressure overload induces an inflammatory myocardium phenotype (Sriramula et al., 2008). Cardiomyocytes can be activated by pressure overload stimuli, resulting in the release of inflammatory cytokines, including interleukin (IL) -1*β*, IL-6, IL-18, and tumour necrosis factor (TNF) -α (Welsh et al., 2009). One investigation showed that IL-1β and the NLRP3 inflammasome play an important role in pressure overload-induced cardiac hypertrophy in mice (Sano et al., 2018). Furthermore, suppressing IL-1β and the inflammasome pathway ameliorates transverse aortic constriction (TAC) -induced ventricular hypertrophy (Zhou et al., 2020). Therefore, anti-inflammatory therapy can be regarded as an important method to prevent cardiac hypertrophy.

Gasdermin D (GSDMD) is the key executioner of pyroptosis, which is a pro-inflammatory programmed cell death (Sborgi et al., 2016). Following inflammasome priming and the cleavage of inflammatory caspases, GSDMD-N gathers to form pores in the cell membrane (Aglietti et al., 2016; Sborgi et al., 2016). These pores destroy the integrity of the cell membrane causing cell swelling and rupture. This process is accompanied by the release of intracellular IL-1β and lactate dehydrogenase (LDH) (Gaidt and Hornung, 2016). Currently, some studies have shown that GSDMD-mediated inflammation also occurs in response to myocardial damage (Yang et al., 2018; Wang et al., 2020a). In addition, Yang F et al. found that inhibiting GSDMD-mediated inflammatory response can effectively alleviate cardiac injury in diabetic cardiomyopathy (Yang et al., 2018). Thus, we hypothesized that pressure overload may induce myocardial hypertrophy through GSDMD and GSDMD-mediated inflammation.

DL-3-n-butylphthalide (NBP) is a lipid-soluble bioactive compound extracted from celery seeds. NBP is widely used in China and other Asian countries to treat ischaemic stroke. It is a neuroprotective agent that significantly reduces ischaemia-induced oxidative damage, inhibits inflammation, regulates energy metabolism, and improves the microcirculation (Abdoulaye and Guo, 2016; Huang et al., 2018). NBP also exhibited potent cardioprotective effects against heart ischaemic injury (Tian et al., 2017), myocardial ischaemia-reperfusion injury (Wang et al., 2014), atrial structural remodelling and atrial fibrillation (Qiu et al., 2018). Despite these promising results, the mechanisms underlying these effects have not been fully identified. In addition, the activity of NBP has not been tested in pressure overload-induced cardiac hypertrophy.

In this study, we challenged mice with TAC to examine the protective effects of NBP. Our studies showed that NBP prevents pressure overload-induced myocardial hypertrophy. We also investigated the effect of NBP on angiotensin (Ang) II-induced cardiomyocyte hypertrophy by utilizing rat primary cardiomyocytes. Furthermore, we discovered that NBP provides these cardioprotective effects by binding to GSDMD and inhibiting GSDMD-mediated inflammation.

## Materials and Methods

### Materials

**FIGURE 1:**
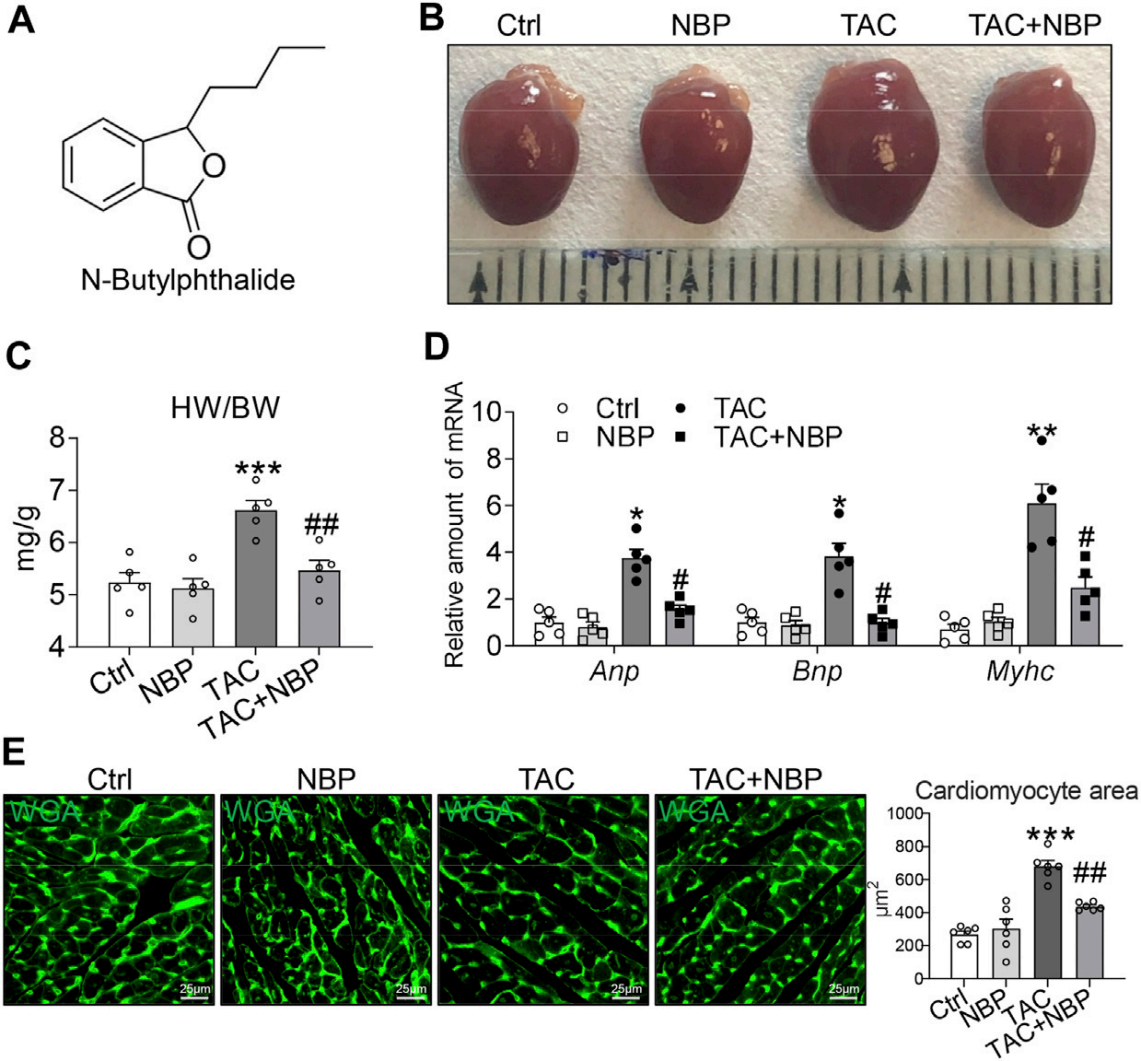
NBP prevents TAC-induced myocardial hypertrophy *in vivo*. **(A)** Chemical structure of NBP. **(B)** Representative gross heart tissues showing the effect of NBP on TAC-induced structural deficits. **(C)** Ratio of the heart weight to body weight (HW/BW). **(D)** mRNA levels of hypertrophy markers Myhc, Anp, and Bnp. **(E)** Representative WGA staining of heart tissues showing the effect of NBP on TAC-induced hypertrophic responses. Quantitative analysis of the cardiomyocyte area is shown in the right panel. (n = 5 in each group; *, vs. Ctrl group; #, vs. TAC group; * and # *p* < 0.05, ** and ## *p* < 0.01, *** *p* < 0.001).

NBP was obtained from CSPC NBP Pharmaceutical C., LTD. with a purity of >99% and dissolved in 0.5% Tween-80 solution for *in vivo* studies (Wang et al., 2014) and dissolved in dimethylsulfoxide (DMSO) for *in vitro* experiments. The chemical structure of NBP is shown in Figure 1A. Ang II was purchased from Sigma (St.Louis, MO, United States). Ang II was dissolved in saline for *in vitro* experiments. Rhodamine Phalloidine was obtained from Solarbio (Beijing, China).

### Animal Experiments

Four-week-old male C57BL/6 mice weighing 18–22 g were obtained from Beijing Vital River Laboratory Animal Technology Co., Ltd. (Beijing, China). All animal care and experimental procedures were performed in accordance with the directives outlined in the Guide for the Care and Use of Laboratory Animals (United States National Institutes of Health). The animal care and experimental protocols were approved by the Committee on Animal Care of The Second Affiliated Hospital of Jiaxing University (Jiaxing, China; approval No. JXEY-2019JX097).

We used a mouse model of transverse aortic constriction (TAC) to induce cardiac hypertrophy. The TAC operation procedures were performed as described previously (Ye et al., 2020). The group of sham operated mice underwent the same procedure without ligation of the aorta (Ye et al., 2020). The NBP treatments were initiated 3 weeks after the mice were subjected to TAC or sham operation. The mice were then maintained on NBP treatment for 5 weeks. NBP was administered by single intraperitoneal injections of NBP at 40 mg kg^−1^ day^−1^ for 5 weeks. That is, we intraperitoneally administered a single injection of 40 mg NBP kg^−1^ every day for 5 weeks. The mice were randomly divided into four groups: the control group (Ctrl, *n* = 5), the NBP group (NBP, *n* = 5, intraperitoneal administration of NBP (40 mg kg^−1^ day^−1^) (Wang et al., 2014; Tian et al., 2017) for 5 weeks), the TAC group (TAC, *n* = 5), and the TAC + NBP group (TAC + NBP, *n* = 5, intraperitoneal administration of NBP in addition to TAC). Eight weeks after TAC, transthoracic echocardiography was conducted on the mice to examine their cardiac function, and then all of the animals were sacrificed under sodium pentobarbital anaesthesia. Blood samples and heart tissues were collected.

### Cardiac Function

Cardiac function was examined in anaesthetized mice noninvasively by transthoracic echocardiography (VisualSonics, Toronto, Canada) 1 day before sacrifice. The left ventricular internal dimension (LVID) in diastole (d), LVID in systolic (s), interventricular septal thickness (IVS)d, IVSs, left ventricular posterior wall thickness (LVPW)d, and LVPWs were assessed from M-mode images. The ejection fraction (EF) was calculated from the LV end diastolic volume (LVEDV) and LV end-systolic volume (LVESV) using the equation (LVEDV-LVESV)/LVEDV*100%. Fractional shortening (FS) was calculated using the equation FS = [(LVIDd- LVIDs)/LVIDd]*100%.

### Wheat Germ Agglutinin (WGA) Staining

Cardiac tissues were collected and embedded in OCT. OCT‐embedded sections were incubated with WGA in phosphate-buffered saline in a dark and humidified container at 37°C for 60 min. Images were taken with a fluorescence microscope (Leica, Germany).

### Cell Culture

Rat primary cardiomyocytes were isolated from Sprague-Dawley (SD) rats as described previously (Wang et al., 2020b) and cultured in DMEM (Gibco, Germany) containing 10% fetal bovine serum, 100 U/ml of penicillin, and 100 U/ml streptomycin.

GSDMD-N overexpression in cells was achieved using GSDMD-N plasmids (GenePharma Co.,Ltd, Shanghai, China). Transfection of primary cardiomyocytes was performed using Lipofectamine 2000 (Invitrogen, Carlsbad, California). Overexpression was verified by western blot.

The CCK8 assay was used to assess the viability of Primary cardiomyocytes according to the manufacturer’s instructions (Nuoyang Biotechnology Co.,Ltd., HangZhou, China).

### Rhodamine Phalloidin Staining

To measure surface area, cells were fixed with 4% paraformaldehyde, permeabilized with 0.1% Triton X-100, and stained with rhodamine phalloidin at a concentration of 50 μg/ml for 30 min. Cells were counterstained with DAPI. Cell surface area was measured using a quantitative digital image analysis system (Image-Pro Plus 6.0)

### Determination of the Lactate Dehydrogenase Levels

LDH levels were measured by an assay kit (Beyotime, Shanghai, China). Media from the primary cardiomyocytes were incubated with LDH working reagent to measure the LDH release according to the manufacturer’s instructions. The heart tissue was incubated with the LDH working reagent to measure the LDH content in the heart.

### Determination of the IL-1β Levels

IL-1β proteins in the media from primary cardiomyocytes were measured by using cytokine-specific ELISA kits (eBiosciences Inc., CA, United States ).

### Real-Time Quantitative PCR (RT-qPCR)

The general procedure for RT-qPCR was described in our previous publication (Han et al., 2018a). Primers for genes including Anp, Bnp, Myhc, and β-actin were synthesized by Invitrogen (Shanghai, China). The primer sequences used are shown in Supplementary Table S1. Target mRNA was normalized to β-actin.

### Western Blot

The general procedure for Western blotting was described in our previous publication (Han et al., 2018a). Antibody against GSDMD-N was obtained from Santa Cruz Biotechnology (Santa Cruz, CA). Antibodies against MyHC and IL-1β were obtained from Abcam (Cambridge, MA, United States). Antibodies against GAPDH and the horseradish peroxidase-conjugated secondary antibody were obtained from Cell Signalling Technology (Danvers, MA, United States).

### Construction of the Initial Complex of GSDMD-N/NBP

The crystal structure of GSDMD-N was obtained from the Protein Data Bank (PDB) database (PDB code: 5B5R) (Ding et al., 2016). The structure of GSDMD-N was processed by the UCSF *Chimaera* program. GSDMD-N and NBP were processed by AutoDockTools. Then, a grid box with a spacing of 0.375 Å was adopted and covered the binding site of GSDMD-N. The binding mode of NBP in the binding site of GSDMD-N was predicted by *AutoDock* (Morris et al., 2009). The conformation with optimal binding energy was selected for subsequent molecular dynamics (MD) simulation analysis.

### Molecular Dynamics Simulation

The GSDMD-N/NBP complex extracted from the molecular docking study was used as the initial structure for the MD simulations. The partial atomic charges for NBP were estimated using the restrained electrostatic potential method. Then, the GSDMD-N/NBP complex was processed using the LEaP module. The complex was then placed into a box of TIP3P water molecules.

First, an equilibration protocol was carried out, including initial minimization, heating, and equilibration of the GSDMD-N-NBP complex. During the minimization steps, 1) solvent atoms were held fixed while solute molecules were relaxed; 2) then solute atoms were held fixed while solvent molecules were relaxed; and 3) both solvent and solute were minimized without any restraints. Eventually, the complex was submitted to 200 ns MD simulation in the NPT ensemble. The parameters applied were described previously (Han et al., 2018b).

### Statistical Analysis

All data are expressed as the means ± sem. Statistical analyses were performed using GraphPad Pro Prism 8.0 (GraphPad, San Diego, CA). Student’s *t*-test, one-way ANOVA followed by multiple comparisons test with Bonferroni correction was used to analyse the differences between sets of data. A *p* value <0.05 was considered significant.

## Results

### Administration of 3-N-Butylphthalide Prevents TAC-Induced Myocardial Hypertrophy *in vivo*

Our first objective was to determine whether NBP inhibits cardiac hypertrophy in mice. To test this hypothesis, we performed TAC to induce cardiac hypertrophic changes. In this model, we initiated NBP administration 3 weeks after performing the TAC operation. The mice were then maintained on NBP treatment for 5 weeks. NBP was administered by single intraperitoneal injections of NBP at 40 mgkg^−1^ day^−1^ for 5 weeks. Gross examination of the harvested hearts showed hypertrophic responses in the TAC mice but not in the mice that received NBP treatment (Figure 1B). The measurement of heart weight normalized to body weight ratios (Figure 1C) was consistent with the result given above. RT-qPCR assays showed that NBP reduced the expression of TAC-challenged pro-hypertrophic genes Myhc, Anp, and Bnp in mouse hearts (Figure 1D). WGA staining of heart tissues showed that NBP attenuated TAC-challenged cardiomyocyte hypertrophy (Figure 1E). These results indicated that NBP prevents TAC-induced myocardial hypertrophy *in vivo*.

### Administration of 3-N-Butylphthalide Protects Against TAC- Challenged Cardiac Dysfunction

**FIGURE 2:**
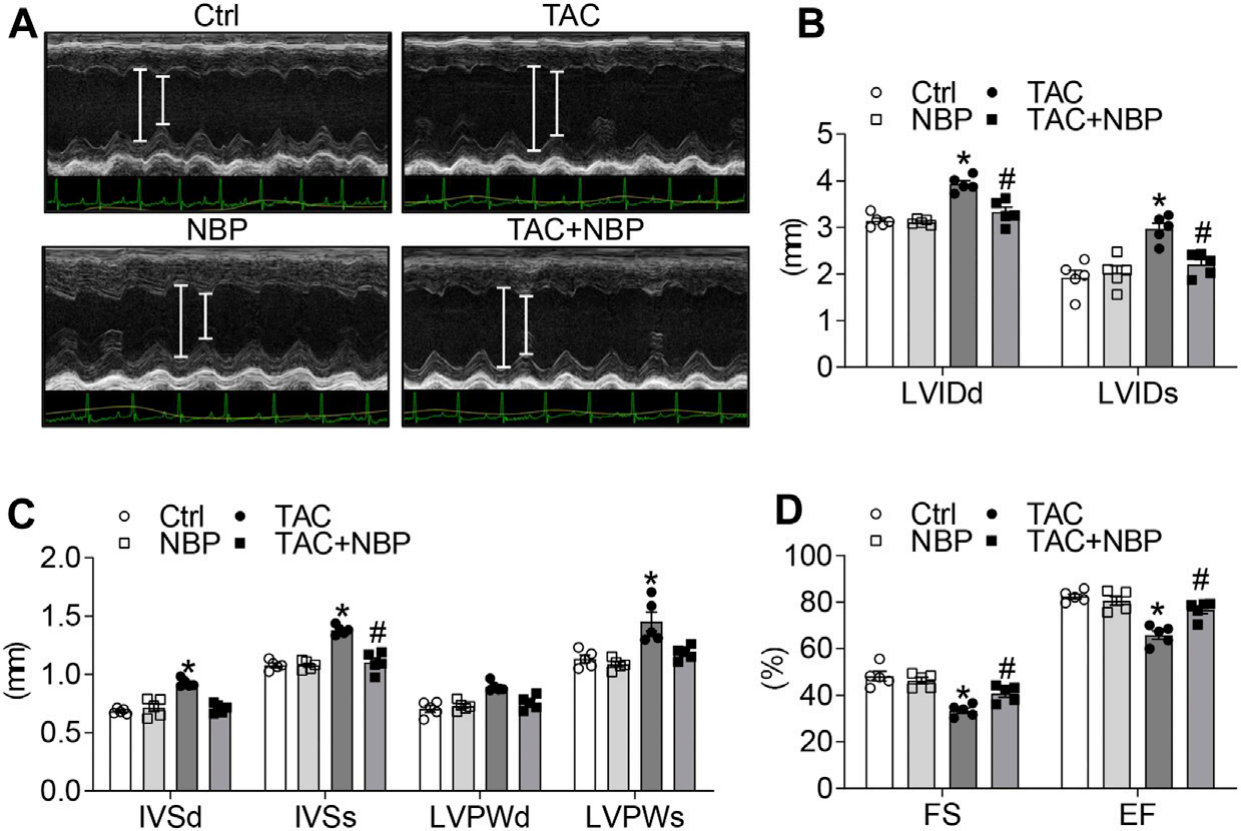
NBP protects against TAC-induced cardiac dysfunction *in vivo*. Echocardiographic data showing the effects of NBP on cardiac dysfunction induced by TAC. **(A)** Representative images of M-mode of left ventricular. **(B)** LVIDd and LVIDs. **(C)** IVSd, IVSs, LVPWd, and LVPWs. **(D)** Ejection fraction (EF); fractional shortening (FS). (n = 5 in each group; *, vs. Ctrl group; #, vs. TAC group; * and # *p* < 0.05).

The cardiac function in the mice was determined by noninvasive transthoracic echocardiography. As shown in Figures 2A,B, the TAC operation increased LVIDd and LVIDs, while NBP administration normalized these alterations. Similar to LVID, NBP treatment decreased IVSd, IVSs, LVPWd, and LVPWs (Figure 2C). Previous studies have shown that the TAC operation decreases EF (%) and FS (%) (Ye et al., 2020). As expected, we found that TAC-induced depression of EF (%) and FS (%) was prevented in mice treated with NBP (Figure 2D). These results demonstrated that administration of NBP prevented TAC-challenged cardiac dysfunction *in vivo*.

### 3-N-Butylphthalide Alleviates Ang II-Induced Cardiomyocyte Hypertrophy and Gasdermin D-Mediated Inflammation

**FIGURE 3:**
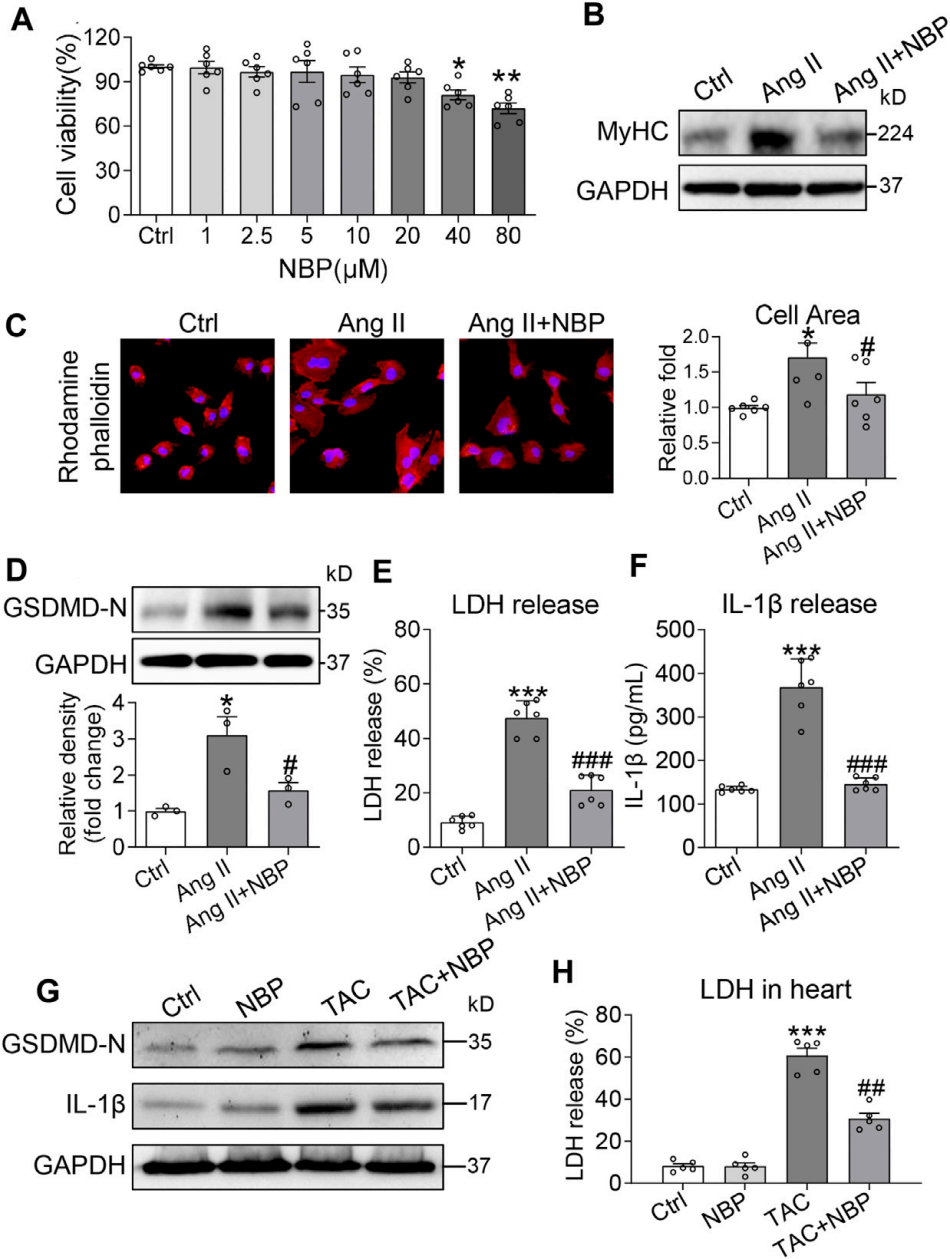
NBP alleviates Ang II-induced cardiomyocyte hypertrophy and GSDMD-mediated inflammation. **(A)** Primary cardiomyocytes were cultured in the presence of the indicated concentrations of NBP for 72 h and then measured for cell viability by CCK-8 assays. DMSO was used as the control. Primary cardiomyocytes were pretreated with NBP (20 μm) for 1 h and then stimulated with 1 μm Ang II for the indicated times. **(B)** After exposure to Ang II for 24 h, cell lysates were probed for β-MyHC by western blot analysis. GAPDH was used as the loading control. **(C)** Rhodamine phalloidin staining of primary cardiomyocytes showing the effect of NBP on Ang II-induced hypertrophy (DAPI: nucleus; × 400 magnification). Quantification of the cell size is shown in the right panel. **(D)** Western blotting analysis of GSDMD in primary cardiomyocytes. The densitometric analysis is shown in the lower panel. **(E–F)** Exposure to Ang II for 24 h showing the LDH and IL-1*β* release from the primary cardiomyocytes. [**A, C, E, F**, n = 6; **(D)**, n = 3; *, vs. Ctrl group; #, vs. Ang II group; * and # *p* < 0.05, ** *p* < 0.01, *** and ### *p* < 0.001] **(G)** Western blotting analysis of IL-1*β* and GSDMD in heart tissues. **(H)** LDH content in TAC mice and mice treated with NBP (n = 5 in each group; *, vs. Ctrl group; #, vs. TAC group; ## *p* < 0.01, *** *p* < 0.001).

Ang II, the main component of the renin-angiotensin-aldosterone system (RAAS), contributes to numerous cardiac pathophysiological processes, including abnormal hypertrophy. Our next objective was to determine whether NBP alleviates Ang II-induced cardiomyocyte hypertrophy. We exposed rat primary cardiomyocytes to 1 μM Ang II. Based on a cell viability assay of NBP at the 72 h time point (Figure 3A), we selected an NBP concentration of 20 μm to assess its effects in cardiomyocytes. Ang II significantly increased MyHC expression in primary cardiomyocytes, while pretreatment with NBP alleviated Ang II-induced expression of this protein (Figure 3B and Supplementary Figure S1A). Furthermore, we performed rhodamine phalloidin staining of primary cardiomyocytes (Figure 3C) and showed that NBP reduced the Ang II-challenged cell size increase in cardiomyocytes. These results indicated that NBP decreased Ang II-challenged hypertrophy in cultured primary cardiomyocytes.

Previous investigations have shown that the IL-1β and NLRP3 inflammasomes are involved in cardiac hypertrophy (Sano et al., 2018; Zhou et al., 2020). The inflammasome is the key priming factor in the canonical pathway of GSDMD activity. Thus, we explored the relationship between NBP and GSDMD-mediated inflammation in Ang II-challenged cardiomyocytes. Our studies showed that Ang II increased GSDMD-N expression in primary cardiomyocytes, and pretreatment with NBP prevented this alteration (Figure 3D). Activated GSDMD-N assembles pores in the plasma membrane, causing cell swelling and extensive release of proinflammatory substances such as IL-1β and LDH. Immunolocalization of GSDMD-N in rat primary cardiomyocytes confirmed GSDMD-N protein translocation from the cytosol to the plasma membrane in the Ang II group (Supplementary Figure S2). Pretreatment of cells with NBP inhibited Ang II-induced GSDMD-N protein translocation (Supplementary Figure S2). In addition, both LDH and IL-1β levels were increased in Ang II-challenged cardiomyocytes, while NBP reduced LDH (Figure 3E) and IL-1β (Figure 3F) release.

To determine whether NBP prevents GSDMD-mediated inflammation *in vivo*, we tested GSDMD-N, IL-1β, and LDH levels in heart tissues of the TAC model. Eight weeks after the TAC operation, we found that TAC induced increased levels of GSDMD-N (Figure 3G and Supplementary Figure S1B), IL-1β (Figure 3G and Supplementary Figure S1B) and LDH (Figure 3H) in heart tissues. Similar to our results in Ang II-challenged primary cardiomyocytes, NBP administration normalized these alterations (Figures 3G,H and Supplementary Figure. S1B).

### Involvement of Gasdermin D in 3-N-Butylphthalide-Mediated Cardiomyocyte Protection

**FIGURE 4:**
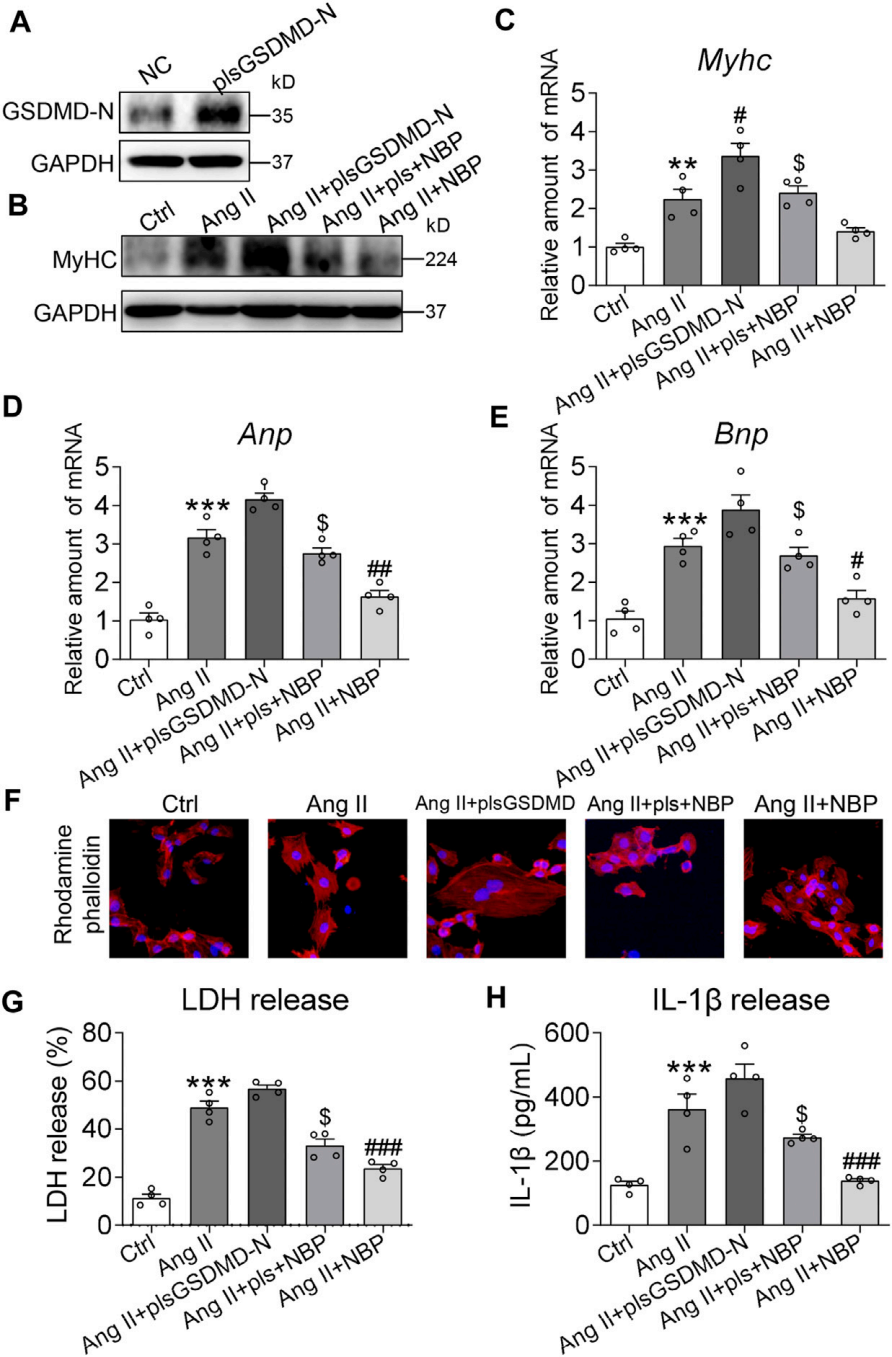
Involvement of GSDMD in NBP-mediated cardiomyocyte protection. **(A)** Primary cardiomyocytes were transfected with cDNA plasmids encoding GSDMD-N (plsGSDMD-N) or empty vector (negative control, NC). Western blotting was performed to assess the protein levels of GSDMD. Primary cardiomyocytes transfected with NC or plsGSDMD-N were pretreated with NBP (20 μm) for 1 h and then exposed to Ang II (1 μm) for the indicated times. **(B)** After exposure to Ang II for 24 h, the effects of NBP and GSDMD-N overexpression on MyHC were detected by western blotting. **(C–E)** After exposure to Ang II for 12 h, the mRNA levels of Myhc, Anp, and Bnp were detected by RT-qPCR. **(F)** Rhodamine phalloidin staining of GSDMD-N-overexpressing primary cardiomyocytes (DAPI: nucleus; × 400 magnification). **(G–H)** After exposure to Ang II for 24 h, LDH and IL-1*β* release were detected by assay kits. (n = 4 in each group; *, vs. Ctrl group; #, vs. Ang II group; $, vs. Ang II + NBP group; # and $ *p* < 0.05, ** and ## *p* < 0.01, *** and ### *p* < 0.001).

To confirm the involvement of GSDMD and its mediation of inflammation in the antihypertrophic effect of NBP in cardiomyocytes, we transfected primary cardiomyocytes with GSDMD-N-expressing plasmids. Transfection of primary cardiomyocytes with specific plasmids (plsGSDMD-N) increased protein levels compared to the negative control (NC) (Figure 4A and Supplementary Figure S1C). For the following *in vitro* studies, we first examined the per se effect of plsGSDMD in contributing to pro-hypertrophic genes and IL-1β release in primary rat cardiomyocytes. The results indicated that plsGSDMD alone did not stimulate an increase in the pro-hypertrophic genes Myhc, Anp, and Bnp or the release of IL-1β (Supplementary Figure S3). Overexpression of GSDMD-N alone produced higher MyHC protein expression in primary cardiomyocytes. Under this saturating condition, NBP failed to reduce the levels of MyHC protein or the NBP + Ang II group (Figure 4B and Supplementary Figure. S1D). RT-qPCR assays of Myhc (Figure 4C), Anp (Figure 4D), and Bnp (Figure 4E) showed the same patterns. Rhodamine phalloidin staining of primary cardiomyocytes confirmed that GSDMD-N overexpression prevents the inhibitory effect of NBP on hypertrophic responses (Figure 4F). We then measured LDH and IL-1β levels in media from primary cardiomyocytes. Pretreatment of NBP of cells transfected with GSDMD-N-expressing plasmids showed less reduction in the levels of LDH (Figure 4G) and IL-1β (Figure 4H) compared with the NBP + Ang II group. Together, the reduced protective effect of NBP on cardiomyocytes following GSDMD-N overexpression suggested that NBP engaged GSDMD in exerting its activities in Ang II‐stimulated primary cardiomyocytes.

### GSDMD-N May Be the Target of 3-N-Butylphthalide in Producing an Anti-inflammatory Phenotype

**FIGURE 5:**
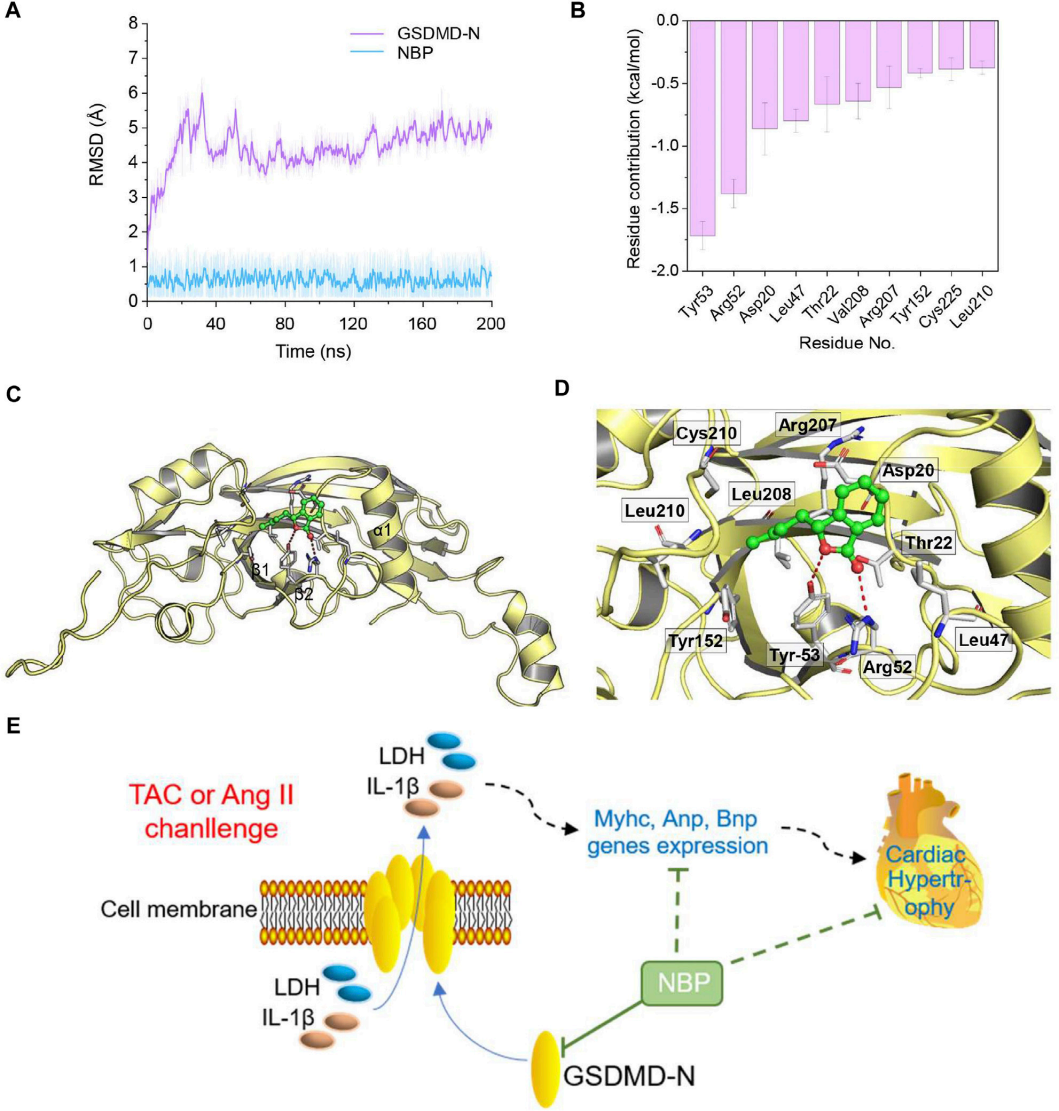
Structural and energetic analysis of NBP to the binding site of GSDMD-N. **(A)** RMSD curves for the 200 ns MD simulation. **(B)** The 10 top contributing residues to NBP bound to GSDMD-N. **(C)** Overview of the predicted binding mode between GSDMD-N and NBP. **(D)** Detailed view of the 10 top contributing residues of NBP bound to GSDMD-N and the hydrogen bonds (red). **(E)** Schematic illustration of the role of NBP in GSDMD-related inflammation in TAC- or Ang II-induced myocardial hypertrophy.

A molecular docking study was performed to predict the possible binding interaction between GSDMD-N and NBP. Then, the optimal complex was submitted to 200 ns MD simulations to explore its dynamic behaviour. The root-mean square deviations (RMSDs) of all of the protein backbone atoms (C_α_) of GSDMD-N and the heavy atoms of NBP were analysed to determine the stability of the studied complex. As shown in Figure 5A, the RMSD values of C_α_ have a small fluctuation after ∼70 ns, and NBP exhibited relative stability during the whole MD simulation. Then, 500 snapshots extracted from the last 40 ns MD simulation trajectory were applied for the subsequent binding free energy decomposition based on the MM/GBSA method. As shown in Figure 5B, the 10 most contributed residues are Tyr53, Arg52, Asp20, Leu47, Thr22, Val208, Arg207, Tyr152, Cys225, and Leu210. Structural analysis indicated that NBP binds to the surface surrounding the α1 helix and β1–β2 sheets in GSDMD-N to disrupt autoinhibition, which provides the primary surface for binding to the GSDMD-C domain (Figure 5C). The primary interactions were hydrogen bonds (Tyr53 and Arg52) (Figure 5D). Overall, the comprehensive simulation methods further verified that GSDMD-N may be the target of NBP in producing an anti-inflammatory phenotype.

## Discussion

This study showed that NBP, which was approved for ischaemic stroke by the State Food and Drug Administration in China in 2002, provides protection against pressure overload-mediated deleterious myocardial hypertrophy by directly binding to GSDMD-N protein and reducing GSDMD-mediated inflammation (Figure 5E). In mice, NBP reduced cardiac hypertrophy and prevented functional deficits in a TAC-induced pressure overload model. In Ang II-challenged cardiomyocytes, we show that NBP treatment prevents cell size increase and inhibits GSDMD-mediated inflammation. Furthermore, overexpression of GSDMD-N reduced the protective effects of NBP against Ang II-induced changes. Using molecular docking and MD simulation, we detected that the GSDMD-N protein may be a target of NBP, indicating that NBP mimics a selective GSDMD inhibitor in preventing cardiac hypertrophy.

NBP, as a neuroprotective agent (Huang et al., 2018; Abdoulaye and Guo, 2016), also exhibited potent cardioprotective effects against heart ischaemic injury (Tian et al., 2017), myocardial ischaemia-reperfusion injury (Wang et al., 2014), atrial structural remodelling and atrial fibrillation (Qiu et al., 2018). In this study, we found that NBP could reduce TAC-induced cardiomyocyte hypertrophy and increase the pro-hypertrophic genes Myhc, Anp, and Bnp (Figure 1). In addition, NBP attenuated cardiac dysfunction and other cardiac functional deficits, such as LVID, IVS, and LVPW, *in vivo* caused by TAC (Figure 2). The myocardial hypertrophy and cardiac dysfunction observed in TAC-induced mice in the current work are consistent with our previous study (Ye et al., 2020).

We used a mouse model of TAC to induce pressure overload-related cardiac hypertrophy as described in our previous study (Ye et al., 2020). The role of the RAAS in many cardiovascular disorders including pressure overload-related cardiac hypertrophy is well established. Ang II is the primary mediator of the RAAS system. Using Ang II stimulation in cardiomyocytes as an *in vitro* experimental model to stimulate TAC- induced cardiac hypertrophy has been reported in our previous study (Ye et al., 2020) and many other researches (Long et al., 2020; Xie et al., 2020). In Ang II-challenged rat primary cardiomyocytes, NBP alleviated the expression of MyHC and increased the size of the cells (Figures 3A–C). These findings indicated that NBP has a protective effect on pressure overload-mediated myocardial damage.

The inflammatory response plays a critical role in the process of cardiac hypertrophy (Freund et al., 2005; Sriramula et al., 2008). Many pro-inflammatory factors are involved in pressure overload-induced cardiac hypertrophy in mice, including IL-1β, IL-6, IL-18, and TNF-*α* (Welsh et al., 2009; Sano et al., 2018). Notably, suppressing IL-1*β* and NLRP3 inflammasome pathways ameliorates TAC -induced ventricular hypertrophy in mice (Zhou et al., 2020). Pyroptosis is a proinflammatory programmed cell death and is regulated by the NLRP3 inflammasome. GSDMD is the key executioner of pyroptotic cell death and it controls the release of LDH and proinflammatory cytokines such as IL-1*β* (Aglietti et al., 2016; Gaidt and Hornung, 2016; Sborgi et al., 2016). Studies have shown that GSDMD-mediated inflammation occurs in myocardial damage (Yang et al., 2018; Wang et al., 2020a). Thus, we hypothesized that pressure overload may induce myocardial hypertrophy through GSDMD and GSDMD-mediated inflammation. Here, we demonstrated that Ang II stimulation and TAC operation increased the expression of GSDMD-N and promoted LDH and IL-1*β* release *in vitro* (Figures 3D–F) and *in vivo* (Figures 3G,H), indicating that GSDMD-mediated inflammation could be used as a molecular mechanism to protect myocardial hypertrophic responses. NBP administration normalized these alterations *in vivo* and *in vitro*.

GSDMD has been reported to be a key executioner in proinflammatory programmed cell death (Nobuhiko et al., 2015; Shi et al., 2015). Here, to further determine the role of GSDMD and its mediation of inflammation in the antihypertrophic effect of NBP in cardiomyocytes, GSDMD-N-expressing plasmids were transfected into rat primary cardiomyocytes (Figure 4A). NBP was not able to inhibit the expression of MyHC (Figure 4B), the cell area increase (Figure 4F), or the release of IL-1β and LDH (Figures 4G,H) in cells transfected with GSDMD-N-expressing plasmids compared with the Ang II + NBP group. These results suggested that NBP may inhibit pressure overload-induced cardiac hypertrophy by blocking GSDMD-N protein and GSDMD-mediated inflammation.

In this study, it is worth noting that we have shown, for the first time to our knowledge, a direct NBP‐binding protein. Using MD simulation, we found a binding conformation of the GSDMD-N/NBP complex, and the 10 most contributed residues were Tyr53, Arg52, Asp20, Leu47, Thr22, Val208, Arg207, Tyr152, Cys225, and Leu210 (Figure 5B). Structural analysis indicated that NBP binds to the surface surrounding the α1 helix and *β*1–*β*2 sheets in GSDMD-N, and the primary interactions involved hydrogen bonds (Tyr53 and Arg52) (Figures 5C,D). Comprehensive simulation methods further verified that GSDMD-N may be the target of NBP in producing an anti-inflammatory phenotype.

This study reveals that NBP, a natural compound, attenuates TAC-induced myocardial hypertrophy and cardiac dysfunction in mice. In addition, NBP engages GSDMD-N protein, a key executioner of pyroptosis, and decreases its expression in cardiomyocytes, which may inhibit the process of GSDMD-N gathering to form pores in the membrane, indicating that targeting GSDMD might be a new strategy for treating myocardial hypertrophy. These findings provide support for the potential use and future research into NBP for the treatment of pressure overload-induced cardiac hypertrophic responses.

## Data Availability

The original contributions presented in the study are included in the article/Supplementary Material, further inquiries can be directed to the corresponding author.
