# Supplementary material for: DL-3-n-Butylphthalide Attenuates Myocardial Hypertrophy by Targeting Gasdermin D and Inhibiting Gasdermin D Mediated Inflammation
Source: Front Pharmacol. 2021 Jun 8;12:688140. doi: 10.3389/fphar.2021.688140 (PMC8217660; doi:10.3389/fphar.2021.688140)
Supplement: Supplementary file 1 [file DataSheet1.DOC]

***Supplementary Information***

**DL-3-n-butylphthalide attenuates myocardial hypertrophy by targeting Gasdermin D and inhibiting it mediated inflammation**

Bingjiang Han^1^,#, Jiajun Xu^1^,#, Xiaowen Shi^1^,#, Zhanxiong Zheng^1^, Fengjie Shi^1^, Fenfen Jiang^1^, Jibo Han^1^,*

1 Department of Cardiology, The Second Affiliated Hospital of Jiaxing University, Jiaxing, Zhejiang, China, 314000

#Bingjiang Han, Jiajun Xu and Xiaowen Shi contribute equally to this paper.

Online Supplement:

1. Supplementary Table S1
2. Supplementary Figure S1-4

**Table S1:** Sequences of primers for RT-qPCR assay used in the study.

| **Gene** | **Species** | **FW** | **RW** |
| --- | --- | --- | --- |
| Myhc | Mouse | CAAAGGCAAGGCAAAGAAAG | TCACCCCTGGAGACTTTGTC |
| Anp | Mouse | ACCTCCCGAAGCTACCTAAGA | CAACCTTTTCAACGGCTCCAA |
| Bnp | Mouse | GTCAGTCGTTTGGGCTGTAAC | AGACCCAGGCAGAGTCAGAA |
| β-actin | Mouse | CCGTGAAAAGATGACCCAGA | TACGACCAGAGGCATACAG |
| Myhc | Rat | GAGGAGAGGGCGGACATT | ACTCTTCATTCAGGCCCTTG |
| Anp | Rat | GGGCTCCTTCTCCATCACC | CTCCAATCCTGTCAATCCTACC |
| Bnp | Rat | CCTAAAACAACCTCAGCCCGT | TTCCGGATCCAGGAGAGACTT |
| β-actin | Rat | AAGTCCCTCACCCTCCCAAAAG | AAGCAATGCTGTCACCTTCCC |


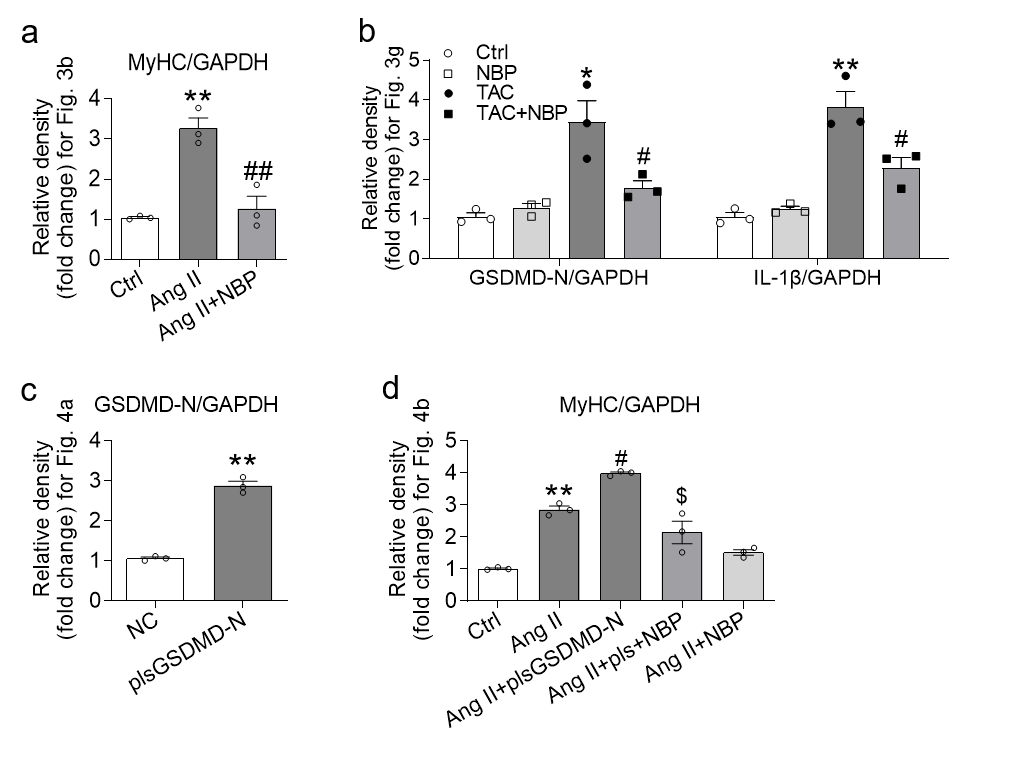


**Figure S1** (a) Densitometric quantification of blots in Figure 3b (n=3; *, vs Ctrl group; #, vs Ang II group; ** and ## P<0.01). (b) Densitometric quantification of blots in Figure 3g (n=3 in each group; *, vs Ctrl group; #, vs TAC group; * and # P<0.05, ** P<0.01). (c) Densitometric quantification of blots in Figure 4a (n=3 in each group; *, vs NC group; ** P<0.01). (d) Densitometric quantifycation of blots in Figure 4b (n=3 in each group; *, vs Ctrl group; #, vs Ang II group; $, vs Ang II+ NBP group; # and $ P<0.05, ** P<0.01).


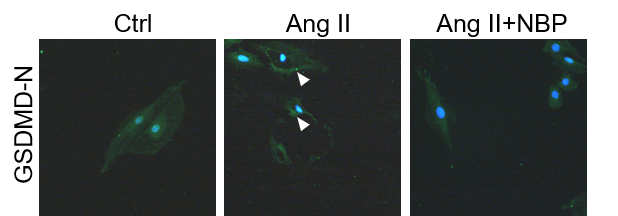


**Figure S2** Immunofluorescence staining of primary cardiomyocytes for GSDMD-N localization (green). Cells were treated as in (Figure 3d) and counterstained with DAPI (blue). Sections were incubated with GSDMD-N antibody. The slides were then incubated with 2 secondary antibodies for 1 h at room temperature. The stained sections were observed using microscope (200× amplification).


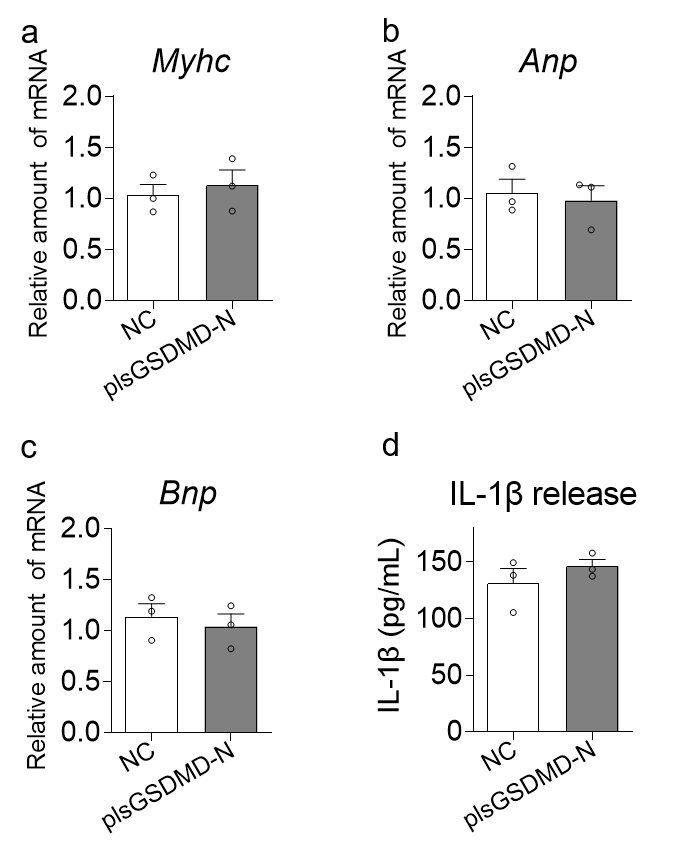


**Figure S3** The per se effect of plsGSDMD on the pro-hypertrophic genes and IL-1β release in primary rat cardiomyocytes. Primary cardiomyocytes were transfected with cDNA plasmids encoding GSDMD-N (plsGSDMD-N) or empty vector (negative control, NC). (a-c) mRNA levels of Myhc, Anp, and Bnp were detected by RT-qPCR. (d) IL-1β release were detected by assay kits. (n=3 in each group)


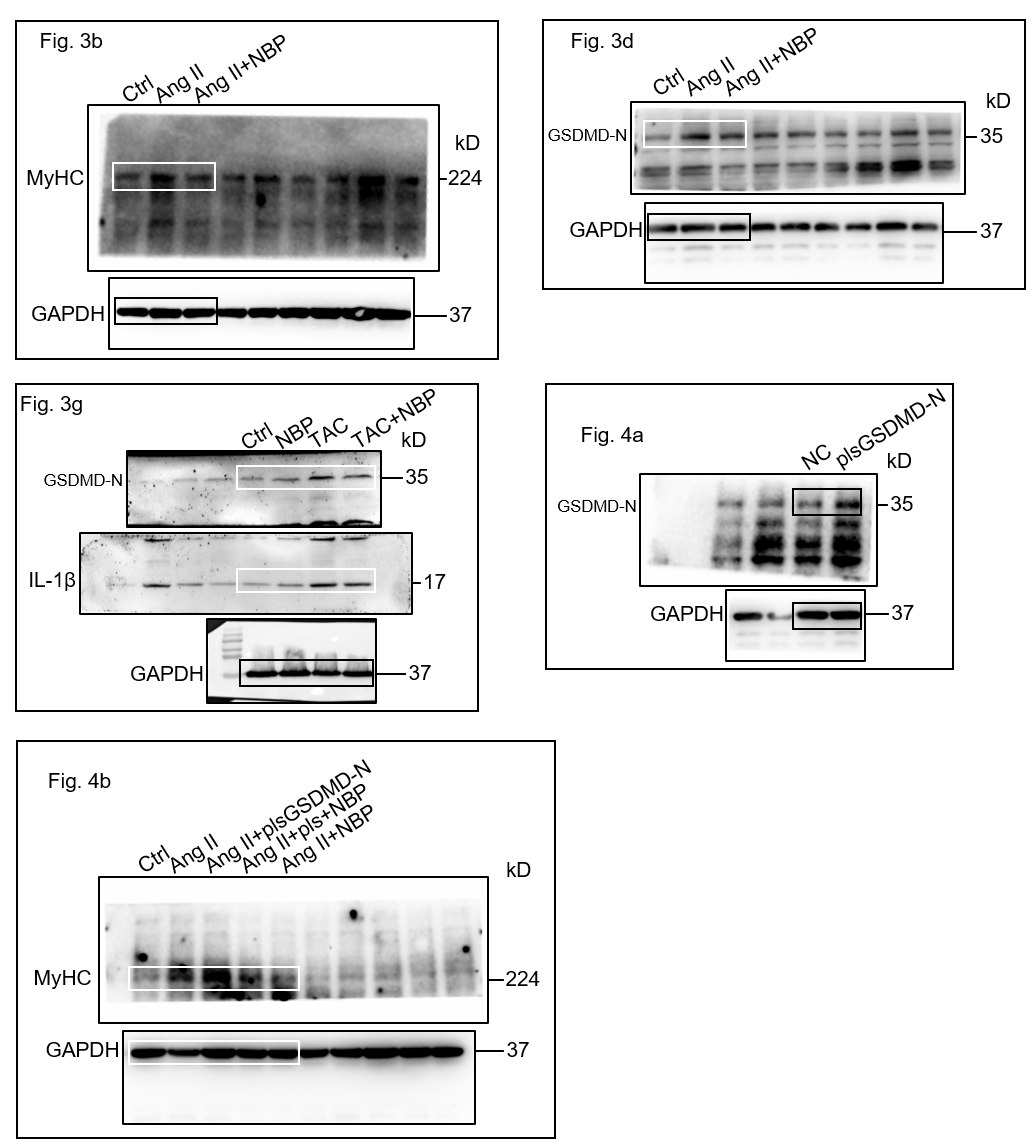


**Figure S4** The original uncut gels of all western blots in this study.
